# The Role of Eph Receptors and Ephrins in Corneal Physiology and Diseases

**DOI:** 10.3390/ijms22094567

**Published:** 2021-04-27

**Authors:** Radoslaw Kaczmarek, Katarzyna Zimmer, Pawel Gajdzis, Malgorzata Gajdzis

**Affiliations:** 1 Department of Ophthalmology, Wroclaw Medical University, 50-556 Wroclaw, Poland; radoslaw.kaczmarek@umed.wroc.pl (R.K.); kasia.zimmer@gmail.com (K.Z.); 2 Department of Pathomorphology and Oncological Cytology, Wroclaw Medical University, 50-556 Wroclaw, Poland; pawel.gajdzis@umed.wroc.pl

**Keywords:** Eph, Eph receptors, ephrin, cornea, corneal neovascularization, epithelium, endothelium

## Abstract

The cornea, while appearing to be simple tissue, is actually an extremely complex structure. In order for it to retain its biomechanical and optical properties, perfect organization of its cells is essential. Proper regeneration is especially important after injuries and in the course of various diseases. Eph receptors and ephrin are mainly responsible for the proper organization of tissues as well as cell migration and communication. In this review, we present the current state of knowledge on the role of Eph and ephrins in corneal physiology and diseases, in particular, we focused on the functions of the epithelium and endothelium. Since the role of Eph and ephrins in the angiogenesis process has been well established, we also analyzed their influence on conditions with corneal neovascularization.

## 1. Introduction

Transmembrane ephrin receptors (Eph) constitute the largest sub-family of tyrosine kinases receptors (RTK). They are widespread in all tissues, especially during embryogenesis and organogenesis. In a mature organism, the expression of Eph in tissues is much lower, except in the nervous tissue [1,2]. Based on their ligand-binding affinity, sequence homology, and structure of the extracellular domain, receptors are divided into two sub-groups. EphA, containing nine receptors (EphA1–A8 and EphA10), and EphB, containing five receptors (EphB1–B4 and EphB6). Unlike other RTKs activated by soluble ligands, Eph interacts with membrane-anchored ephrins, also divided into two subtypes—ephrinA (A1–A6) and ephrinB (B1–B3) [3,4]. EphrinsA are tethered to the extracellular cell membrane via a glycosylphosphatidylinositol (GPI) anchor. EphrinsB are transmembrane proteins with a short cytoplasmic region containing a PDZ-binding motif [2]. EphA binds preferentially to ephrinsA and EphB to ephrinsB. However, there are exceptions to this rule, for example, EphA4, which reacts with both ephrinsA and ephrinsB [2].

Due to their close bond with the cell membranes, Eph and ephrins can perform their basic functions, which is positioning cells and organizing their layers in relation to each other and surrounding tissues during embryogenesis, additionally controlling migration, adhesion, repulsion, and differentiation of cells in the mature organism [3,5]. They play an important role in angiogenesis, vasculogenesis, and axon guidance [1,6]. They are responsible, among other things, for the formation of the main blood vessels, valves, and septum in the heart. They also participate in the development of lymphatic vessels. Moreover, Eph receptors mediate cell-to-cell interactions not only in cells, but also in the microenvironment—stroma and vasculature [2,7]. Eph and ephrins are directly or indirectly responsible for a number of processes in a healthy body, which together can be called the plasticity of mature tissues. They play a key role in maintaining homeostasis of epithelial tissues, especially in the breast, intestines, and skin [8]. In the circulatory system, they support communication between endothelial cells, smooth muscle cells, and pericytes, stabilizing the walls of arteries and veins and regulating the penetration of monocytes through them [3,9]. Their role in the platelet aggregation also has been proven [10]. They participate in the arterial pressure regulation and they affect the function of the cardiac progenitor cells, which are responsible for the heart muscle regeneration [11]. Considering the nervous system, Eph and ephrins play an important role in the processes of learning, memory, and synaptic plasticity, as well as in the nervous tissue regeneration after injuries and ischemia attacks [2,11]. All the functions of Eph and ephrin described above can be performed thanks to the modulation of cell-cell adhesion, deadhesion, and retraction. The type of response to the Eph/ephrin system depends on many factors, such as the intensity of the expression of receptors and ligands on the cell surface, composition of signaling clusters, receptor cis or trans configuration, degree of oligomerization, disruption and internalization of Eph/ephrin complexes, participation of various regulatory molecules, and cross-talk with other cell signaling systems [3].

The cornea is located in front of the eye. Anatomically, it is an extension of the sclera. The human cornea consists of the five layers. The first, outer layer is the stratified, nonkeratinizing squamous epithelium [12]. Together with the tear film that overlays them, they create a protective barrier against infectious, chemical, and toxic agents and injuries. Epithelial cells create tight junctional complexes between each other, which allow them to maintain their proper function as a protective barrier, as well as create a refractive surface ensuring correct vision [13]. Bowman’s membrane, an acellular layer, separates the epithelium from the stroma. The stroma consists mainly of collagen bundles surrounded by proteoglycans. The dominant type of cells located between collagen fibers are keratocytes. The strictly defined organization of the stroma structure reduces forward light scatter, ensures the transparency of the tissue, and maintains it adequate strength and flexibility [14]. The innermost layer of the cornea is the endothelium, separated from the stroma by the Descemet’s membrane. The single-layer endothelium, composed of the hexagonal cells, accounts for the corneal clarity by keeping it in a relatively dehydrated condition [15].

The cornea is an unusual tissue that is characterized by perfect transparency, strength, and a strictly defined shape. It protects the inside of the eye against injuries and infections and constitute an essential element of the optical system that allows proper vision [12]. To maintain its properties, the perfect organization of individual cells is extremely important. Due to exposure to the external factors, it must also be able to recover quickly and effectively after superficial injuries [16]. Knowing the basic functions of Eph and ephrin, it can be deduced that they are involved in the functioning of the cornea.

## 2. Eph and Ephrin Expression in the Healthy Cornea

The expression of Eph and ephrin in a physiological cornea has not been carefully investigated, yet. However, determining the expression profile of the receptors and their ligands in the healthy tissues is essential for understanding their role in the physiological processes as well as in the disease development. In most studies, the expression of Eph receptors and ephrins is determined in vivo experiments in tissues and in cell lines. Results vary, and often a greater variety of receptors is found in the cell lines. The reason for this is not fully understood. The main hypothesis states that receptors become active at certain phases of the cell cycle. Another one is that receptors that are highly species-specific may not react with certain types of animal-derived antibodies used in the investigations. The exact explanation of this phenomenon requires much further investigation and intensive research. Kojima et al. investigated the expression of all up to date known Eph receptors and their ligands in mice corneas. They examined not only healthy tissues but also corneas with induced neovascularization [17]. According to the published results, healthy corneal epithelium expresses EphA3. This is an interesting observation as EphA3 is also expressed in the ophthalmic trigeminal nerve. This may indicate the involvement of EphA3 in trigeminal nerve guidance during nerve reinnervation or maintenance [18]. The remaining receptors found in healthy corneas were EphB1, located mainly in the epithelium and in keratocytes, and EphB4, which was expressed mainly in the epithelium and stroma [16].

**Figure 1:**
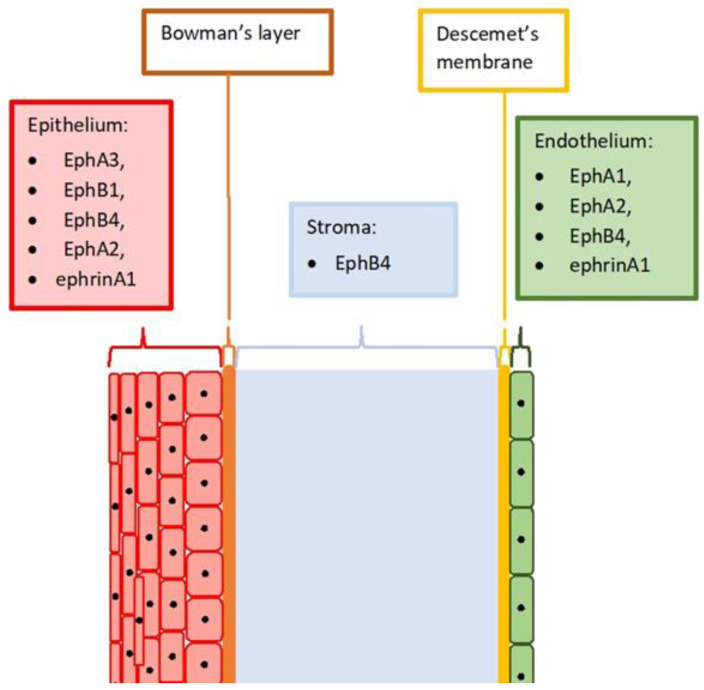
Diagram of a healthy cornea. Next to the names of the individual layers, the Eph receptors and ephrins are mentioned, the presence of which has been known so far.

In the study on human corneas, Walshe et al. showed that EphA1 and EphA2 are expressed in the corneal endothelial cells [19]. Hogerheyde et al. proved that the healthy endothelium expresses also ephrinA1 and EphB4. EphB4 was also present in the epithelium and in the cells located between the collagen lamellae of the peripheral stroma. The most intense EphB4 immunoreactivity was detected at the junctions between the individual epithelial cells [20]. Kojima et al.’s study also showed that EphA2 is present at the cell-cell borders throughout all layers of the corneal epithelium [21]. These observations confirmed the significance of the Eph in maintaining the integrity of tissues and the organization of the cells in relation to each other. Figure 1 shows a schematic drawing of the cornea. The known expression of Eph and ephrins in individual layers is described.

## 3. Effect of Eph and Ephrins on the Corneal Epithelium

Stratified nonkeratinized epithelium is an outer barrier that protects underlying tissues from the external environment, injuries, and infections. The ability to quickly and efficiently repair epithelial defects is particularly important [22]. After the injury, stem cells migrate from the basal layers of the limbus towards the damage. The process of the repair at the molecular level is complex. The role of Eph receptors and ephrins in the healing of the corneal epithelium is relatively still poorly understood [23]. Research published so far focuses mainly on EphA2 and ephrinA1. Kaplan et al. showed that ligand activation of EphA2 restricts the migration of corneal epithelial cells [21]. They also hypothesized that pathological conditions that increase the expression of ephrinA1, a ligand for EphA2, may cause disturbances in the cell migration and, consequently, impair wound healing. Diabetes is an example of such a condition. The healing processes of the corneal epithelium in diabetic patients are impaired compared to those in healthy people [24]. Many factors are responsible for this state, including reduced Akt and Erk1/2 phosphorylation [25]. These reactions occur as a result of EphA2 activation by ephrinA1. Further research is essential to fully understand the effects of Eph and ephrin on the corneal epithelium healing. However, in vitro studies have shown that elevated glucose levels induced an elevated ephrin A1 expression, which eventually decreased EphA2 levels and impaired migration of epithelial cells [21]. Moreover, cell lines with increased EphA2 expression and treated with exogenous ephrin-A1-Fc, exhibited slower wound healing and reduced cell migration. Treatment of primary human corneal epithelial keratinocytes isolated from corneoscleral rims with ephrin-A1-Fc also decreased wound healing. This reduction was due to the inhibition of Akt signaling, a well-studied mediator of epithelial cell proliferation and migration [21].

At the border of the cornea and limbus, the structure of the epithelium changes. The more differentiated corneal epithelial basal cells are clearly segregated from limbal, which are enriched in progenitor cells [22]. There are also significant differences in the expression of the certain proteins, such as enolase, calcium-linked epithelial differentiation protein (CLED), and early epithelial differentiation protein (EEDA) [26]. Maintaining the correct organization of the cells in physiological conditions and restoring it after injury is significant in maintaining the proper function of the corneal epithelium [27]. In the case of improper regeneration, when the cells do not adhere tightly to each other and to the Bowman’s membrane, spontaneous epithelial defects appear. They are very painful, open the door to potential infection, and worsen the optical parameters of the cornea. Kaplan et al., in their studies, showed that EphA2 and ephrinA1 play a key role in limbal–corneal epithelial compartmentalization. They prevent the migration of epithelial cells into the fields with high levels of ephrin ligands, helping maintain normal tissue compartmentalization in the conjunctiva, limbus, and cornea [26]. 

Another example of disturbed cell migration in the surface epithelium of the eye is pterygium. Pterygium is a type of benign growth of connective tissue that situates above the sclera. It arises mainly as a result of direct exposure to UV radiation. The formation of pterygium leads to uncontrolled proliferation of cells. The lesion is classified as a mild one, but the disease frequently reoccurs [28]. John-Aryankalayil et al. showed that in the pterygium tissues, the level of ephrinA1 is reduced, which probably disrupts the organization of the tissues and provokes invasion of the surrounding tissues [29].

The processes of fibrosis and angiogenesis play an important role in the pathogenesis of pterygium. The vascularization within the pterygium is much more intense than in the physiological conjunctiva. Blood vessels morphology is typical for neovascularization—they are smaller in diameter and more branched. The vascular microdensity analysis shows that in the pterygium, intense angiogenesis processes appear, and the expression of VEGF is also increased [30]. Several studies have confirmed the role of Eph receptors in angiogenesis in the pterygium development. Xue et al. showed that pterygium tissue compared to normal conjunctiva showed high EphB4 expression and higher average count of microvessels. The density in the examined tissues showed a significant correlation with the expression of EphhB4 [31]. The same authors in another study showed that EphB4 and ephrinB2 are expressed throughout the pterygium epithelium, while in the healthy conjunctiva, they are found only in the basal layers [32].

## 4. Effect of Eph Ad Ephrins on the Corneal Endothelium

The endothelium is primarily responsible for maintaining the transparency of the cornea [33]. It consists of a monolayer of cells arrested mainly in the G1 phase of the cell cycle [20]. Endothelial cells have no mitotic activity in vivo [12]. Hogerheyde et al. showed that the corneal endothelium expresses ephrinA1 [20]. EphrinA1 was found mainly in the cytoplasm of endothelial cells and exhibited immunoreactivity evenly over the entire surface, both in the center and at the periphery of the cornea. Since ephrinA1 is a potent inhibitor of cell proliferation, in particular of the vessels endothelium, it can be assumed that it may be partly responsible for the arrest of cells in particular phase of the cell cycle [34]. Further detailed studies are needed to see if modulation of Eph and ephrin-related signaling pathways could help in the development of corneal endothelial regeneration strategies [35].

**Figure 2:**
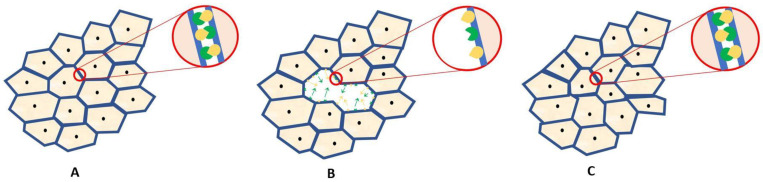
Diagram of the healing process of the corneal endothelium. (**A**) A healthy endothelium is made up of a single layer of hexagonal cells tightly adjacent to each other. (**B**) When cells are lost, spaces are created in the endothelial layer. The surrounding cells begin to migrate towards the lesion (green and yellow arrows). (**C**) When the healing process is complete, cells reattach to each other but lose their hexagonal shape and exhibit variability in size. Membrane-bound Eph and ephrins are expected to contribute to cell adherence and migration.

The integrity of the endothelium layer is essential for proper functioning. Any breakdown can result in the corneal oedema, and consequently, serious vision impairment. When some cells are lost due to aging, injury, or disease, the remaining cells fill in the gaps by spreading and migrating, growing and losing their hexagonal shape (Figure 2) [12]. Walshe et al. investigated whether Eph/ephrins had an effect on endothelial cell motility. They demonstrated that cultures of the corneal endothelial cells display reduced migration and reduced levels of E-cadherin mRNA, following suppression of ligand-activated Eph receptor signaling. They used lithocholic acid, a reversible, competitive, and non-selective antagonist of the Eph/ephrin system, to reduce the migration of corneal endothelial cells in the in vitro wound scratching test [19]. The research results are interesting, but it should be remembered that lithocholic acid, a secondary bile acid, acts as a regulatory molecule, reacting with many receptors, for example, the nuclear farnesoid X receptor (FXR) and the G protein coupled TGR5 receptor [36]. For this reason, the obtained results require careful checking in subsequent tests. However, the above studies indicate that Eph and ephrins may have a significant influence on the maintenance of the normal function of endothelial cells.

## 5. Corneal Neovascularization

Corneal neovascularization (NV) is a significant problem in ophthalmic practice. The essence of the disease is the ingrowth of the blood vessels into the tissue, which under normal conditions should remain avascular. Corneal avascular state, also defined as corneal angiogenic privilege, is a complex process involving the maintenance of the balance between anti-angiogenic and proangiogenic factors. This balance is shifted towards proangiogenic factors with every injury, but the healing process does not always lead to the development of NV [37]. Untreated NV could lead to further complications such as oedema, lipid deposition, and tissue scarring, resulting in a significant deterioration of visual acuity and quality of life [38]. Exact epidemiological statistics are not yet known. One study showed that up to 4% of ophthalmic patients in the US show symptoms of NV. It is also known that 20% of the samples taken during the corneal transplant procedure show signs of NV on histopathological examination [39].

Corneal NV is usually associated with inflammatory or infectious disorders of the ocular surface. One of the most common infectious causes of corneal NV is herpes simplex virus infection. In developed countries, the incidence is estimated at 5.9–20.7 per 100,000 person/year. Prevalence is approximately 149 per 100,000 person/year [39]. Most often, NV is associated with a recurrent form of the disease affecting the stroma of the cornea. Unfortunately, its incidence and prevalence are unknown [40]. Other infections that often lead to NV are trachoma and onchocerciasis. Trachoma, caused by infection with Chlamydia trachomatis, is responsible for over 6 million cases of blindness worldwide, the vast majority related to NV. Onchocerciasis, a parasitic infection, accounts for approximately 1 million total blindness cases in tropical countries, all associated with corneal NV [40].

Another frequent cause of the corneal NV are complications with wearing contact lenses. Corneal NV affects 1–20% of contact lens wearers. The vast majority of this applies to soft lenses, especially in extended wearers. Moreover, 10–30% of patients diagnosed with corneal neovascularization wear contact lenses [41]. The most likely cause is the exposure of the cornea to chronic hypoxia. Other possible causes are chronic direct mechanical damage to the surface of the eye in the event of a poor fitted lenses, or disturbance of the tear film due to bad biocompatibility [42].

Other less common causes of corneal NV are bacterial infections, chemical burns (especially alkali burns), trauma, degenerations (e.g., pterygium and Terrien marginal degeneration), aniridia, and inflammatory conditions such as graft rejection, rosacea, pemphigoid, and Steven’s Johnson syndrome [39].

There are two main types of corneal invading vessels. The conjunctival vessels, responsible for the superficial vascularization, are bright red and well defined. They tend to branch and may raise irregular epithelium. They cross the limbus and run in the superficial layers of the stroma. Vessels sprouting from the capillaries and venules of the pericorneal plexus, made up of the branches of the ciliary arteries, are responsible for deep NV. They run in profound stromal layers, disappearing at the limbus and cannot raise epithelium. They are dark red, ill-defined, and run parallel and radially [39].

In clinical practice, there are three basic types of the corneal NV [43]. In superficial vascularization, vessels extend beneath the epithelium. They sprout from the superficial marginal arcade. This type of NV develops in response to corneal trauma, mild chemical burns, inflammation, and infections [40]. The second type of NV within the cornea is pannus formation. The pannus is composed of connective tissue proliferating in the superficial corneal periphery. Collagen and vessels grow from the limbus onto the peripheral cornea and can cause permanent scarring [39]. Pannus formation is mainly associated with disorders of the surface of the eye, especially when irritants act on the cornea for a long time [37]. Lastly, stromal vascularization can occur at any level of the stroma, from beneath Bowman layer to Descemet membrane. It is associated with most forms of stromal keratitis [37]. Deep NV, overlying Descemet’s membrane can be seen in serious anterior segment injuries, tuberculosis, syphilis, and scleritis [39].

Abnormal vessels within the cornea arise as a result of angiogenesis, the sprouting and branching of new vessels from the existing ones. In the process of angiogenesis, the most important are VEGF/VEGF receptor, angiopoietin/Tie2, and ephrin/Eph receptor complexes [44]. Eph and ephrins are involved in the angiogenesis process at every stage, starting with proliferation and migration of endothelial cells, assembly and recruitment of perivascular cells, and finally remodeling of the extracellular matrix [45,46,47].

## 6. Eph and Ephrin Expression in Neovascularized Corneas

Under NV conditions, the corneal tissues express more Eph receptors and ephrins than the healthy tissues (Table 1).

The already mentioned study by Kojima et al. showed the expression of EphA3, ephrinA1, EphB1, EphB4, and ephrinB1 in the epithelium of mice cornea with NV (ex vivo study) [17]. EphrinB1 and EphB1 were also immunolocalized to the stroma of the vascularized cornea, and their distribution was strongly related to the vessels. EphrinB1 was immunolocalized mainly to the keratocyte around the vascular endothelium and EphB1 was localized in the endothelium. In another study, EphA3 was immunolocalized in mouse corneal epithelial cells, keratocytes, and stroma [44]. EphA2 was immunolocalized only in the epithelial cells. EphA1 and ephrinA2 were detected in the epithelial cells and keratocytes. In addition, ephrinA1, EphB1, EphB4, ephrinB1, and ephrinB2 were detected in the corneal epithelial cells, keratocytes, and stroma. In the vascularized corneas, ephrinB1 was mainly immunolocalized to the keratocytes around the vessels, and ephrinB2, EphB1, and EphB4 were mainly localized in the vascular endothelial cells. The above observations seem to confirm the role of Eph and ephrin in the development of NV within the cornea. The location of receptors within the endothelium and their ligands in keratocytes around the endothelium is particularly noteworthy. This observation is consistent with the mechanism of action of Eph and ephrins described in the literature, which increases the reliability of the results.

## 7. Eph and Ephrins in Corneal Neovascularization Models

The experimental models developed for the needs of the clinical trials prove the role of Eph and ephrin in the corneal NV process. Pellets impregnated with test substances are implanted into micropockets in the cornea, using animals, most often mice and rats, in the research [48]. Such studies have shown that ephrinB2 induces angiogenesis in vivo and newly formed vessels infiltrated the pellet [49]. Another, more detailed study showed that ephrinB2 induces NV in the cornea and that the newly formed vessels are weaker but of similar length compared to the VEGF-induced ones. Comparing the blood vessels formed under the influence of ephrinB2 and VEGF, it was found that ephrinB2 has a stronger effect on the formation of venous vessels, while VEGF induces the formation of arteries [50]. Activation of EphB1 also induces angiogenesis within the cornea, as does ephrinB1 and ephrinB2 [44,51]. Additionally, ephrinA1 induced corneal NV in the experimental models [46,52]. Moreover, Cheng et al., in studies on the corneas of mice, proved that EphA receptor activation is necessary for VEGF-induced angiogenesis and the soluble form of the EphA2 receptor inhibits ephrinA1-induced corneal angiogenesis in vivo [46].

## 8. Possible Applications of Eph and Ephrin Signaling System Modifying Drugs in Corneal Diseases

In the above considerations, we have shown that Eph and ephrins play an important role in various corneal diseases. This makes them a potentially interesting target for therapy. However, their safety profile must be confirmed. Some of the systemic drugs in the treatment of cancer have an affinity for Eph receptors. For example, dasatinib, a multi-targeted kinase inhibitor, potently inhibiting EphA2 and other Eph receptors [53]. Based on their characteristics, preliminary conclusions about side effects can be drawn. There are also several clinical trials in which patients are given systemic EphB inhibitors (clinical trial numbers: NCT02717156; NCT02767921; NCT03049618; NCT03146971; NCT02495896; NCT01642342; NCT03049618). The results have yet to be published, but none of the studies have been discontinued due to unacceptable toxicity.

However, it should be remembered that in ophthalmology, drugs are administered mainly topically, therefore, the potential toxicity to the structures of the eyeball will be of the decisive importance. Several studies have been published in which EphB inhibitors were administered by intravitreal injections to mice and rats [54,55,56,57]. In these studies, intravitreal administration of Eph inhibitors inhibited retinal NV in animal models. The reduction in NV was demonstrated by immunohistochemistry. There were no necrotic changes or other deviations from the norm that could indicate the toxicity of the tested substances in the examined fragments of the retina. Brar et al., in rabbit studies, showed that the intravitreal administered soluble form of the EphB4 receptor (sEphB4) showed no toxicity to the ocular tissues [58]. As the structures inside the eyeball, in particular, the retina, are more susceptible to damage caused by drugs, the above results suggest that also topically applied drugs should not be toxic to the conjunctiva and the cornea [59]. In drug research, however, it should be remembered that both their bioavailability and possible toxicity may vary significantly depending on the species under study. For this reason, even very good results in the treatment of animals do not necessarily mean success for humans [60]. Due to the specific structure of the cornea, the route of administration is also important for the final result. For the treatment of the cornea, it is usually necessary to administer drugs topically in the form of drops [61]. With this route of administration, due to the rich vascularization of the conjunctiva, relatively more substances are absorbed into the bloodstream [62].

More and more studies are available on drugs modifying the action of Eph and ephrins [63,64]. Much attention is paid to the role of monoclonal antibodies targeting Eph receptors, due to their relatively high target specificity [65,66]. Of course, the exact safety profile of Eph inhibitors still requires intensive research. Hopefully, more centers will test them over time for treating eye diseases as preliminary results are very promising.

## 9. Summary

Eph and ephrins form an extremely diverse and interesting group. Due to the already known functions, they are the subject of many studies, not only in the context of oncology. The cornea seems to be an inconspicuous structure, but it should be remembered that even the slightest disturbance in its functioning can lead to serious complications—a significant deterioration of vision, as well as pain in patients. What is worse, due to its complicated structure and the need to maintain translucency, the therapeutic possibilities are very limited. There are currently more questions than answers regarding the relationship between Eph and ephrin and corneal diseases. However, it is certainly worth conducting further research in order to be able to use the acquired knowledge in the effective treatment of patients in the future.

## Figures and Tables

**Table 1:** Comparison of Eph and ephrin expression in normal and NV cornea.

	Healthy Cornea	NV Cornea
EphA1	Endothelium	Epithelium
EphA2	Epithelium, endothelium	Epithelium
EphA3	Epithelium	Epithelium, stroma
EphB1	Epithelium	Epithelium, stroma
EphB4	Epithelium, stroma, endothelium	Epithelium, stroma
ephrinA1	Epithelium, endothelium	Epithelium, stroma
ephrinA2	Not shown	Epithelium, stroma
ephrinB1	Not shown	Epithelium, stroma
ephrinB2	Not shown	Epithelium, stroma

## References

[1] Pasquale E.B. (2008). Eph-Ephrin Bidirectional Signaling in Physiology and Disease. Cell.

[2] Xi H.-Q., Wu X.-S., Wei B., Chen L. (2012). Eph receptors and ephrins as targets for cancer therapy. J. Cell. Mol. Med..

[3] Nievergall E., Lackmann M., Janes P.W. (2012). Eph-dependent cell-cell adhesion and segregation in development and cancer. Cell. Mol. Life Sci..

[4] Surawska H., Ma P.C., Salgia R. (2004). The role of ephrins and Eph receptors in cancer. Cytokine Growth Factor Rev..

[5] Rüegg C., Mariotti A. (2003). Vascular integrins: Pleiotropic adhesion and signaling molecules in vascular homeostasis and angiogenesis. Cell. Mol. Life Sci..

[6] Cramer K.S., Miko I.J. (2016). Eph-ephrin signaling in nervous system development. F1000Research.

[7] Ogawa K., Pasqualini R., Lindberg R.A., Kain R., Freeman A.L., Pasquale E.B. (2000). The ephrin-A1 ligand and its receptor, EphA2, are expressed during tumor neovascularization. Oncogene.

[8] Perez White B.E., Getsios S. (2014). Eph receptor and ephrin function in breast, gut, and skin epithelia. Cell Adh. Migr..

[9] Pfaff D. (2008). Involvement of endothelial ephrin-B2 in adhesion and transmigration of EphB-receptor-expressing monocytes. J. Cell Sci..

[10] Tognolini M., Hassan-Mohamed I., Giorgio C., Zanotti I., Lodola A. (2014). Therapeutic perspectives of Eph–ephrin system modulation. Drug Discov. Today.

[11] Barquilla A., Pasquale E.B. (2015). Eph receptors and ephrins: Therapeutic opportunities. Annu. Rev. Pharmacol. Toxicol..

[12] DelMonte D.W., Kim T. (2011). Anatomy and physiology of the cornea. J. Cataract Refract. Surg..

[13] Patel S., Tutchenko L. (2019). The refractive index of the human cornea: A review. Contact Lens Anterior Eye.

[14] Boote C., Dennis S., Newton R.H., Puri H., Meek K.M. (2003). Collagen fibrils appear more closely packed in the prepupillary cornea: Optical and biomechanical implications. Investig. Ophthalmol. Vis. Sci..

[15] Stiemke M.M., Edelhauser H.F., Geroski D.H. (1991). The developing corneal endothelium: Correlation of morphology, hydration and na/k ATPase pump site density. Curr. Eye Res..

[16] Meek K.M., Knupp C. (2015). Corneal structure and transparency. Prog. Retin. Eye Res..

[17] Kojima T., Chung T.Y., Chang J.H., Sayegh R., Casanova F.H., Azar D.T. (2007). Comparison of EphA receptor tyrosine kinases and ephrinA ligand expression to EphB-ephrinB in vascularized corneas. Cornea.

[18] Jayasena C.S., Flood W.D., Koblar S.A. (2005). High EphA3 expressing ophthalmic trigeminal sensory axons are sensitive to ephrin-A5-Fc: Implications for lobe specific axon guidance. Neuroscience.

[19] Walshe J., Richardson N.A., Al Abdulsalam N.K., Stephenson S.A., Harkin D.G. (2018). A potential role for Eph receptor signalling during migration of corneal endothelial cells. Exp. Eye Res..

[20] Hogerheyde T.A. (2013). Evaluation of Eph receptor and ephrin expression within the human cornea and limbus. Exp. Eye Res..

[21] Kaplan N., Fatima A., Peng H., Bryar P.J., Lavker R.M., Getsios S. (2012). EphA2/ephrin-A1 signaling complexes restrict corneal epithelial cell migration. Investig. Ophthalmol. Vis. Sci..

[22] Lavker R.M., Kaplan N., Wang J., Peng H. (2020). Corneal epithelial biology: Lessons stemming from old to new. Exp. Eye Res..

[23] Zhu L., Titone R., Robertson D.M. (2019). The impact of hyperglycemia on the corneal epithelium: Molecular mechanisms and insight. Ocul. Surf..

[24] Shih K.C., Lam K.L., Tong L. (2017). A systematic review on the impact of diabetes mellitus on the ocular surface. Nutr. Diabetes.

[25] Xu K., Yu FS X. (2011). Impaired epithelial wound healing and EGFR signaling pathways in the corneas of diabetic rats. Investig. Ophthalmol. Vis. Sci..

[26] Kaplan N. (2018). Epha2/ephrin-A1 mediate corneal epithelial cell compartmentalization via ADAM10 regulation of EGFR signaling. Investig. Ophthalmol. Vis. Sci..

[27] Lavker R.M., Tseng S.C., Sun T.T. (2004). Corneal epithelial stem cells at the limbus: Looking at some old problems from a new angle. Exp. Eye Res..

[28] Liu T., Liu Y., Xie L., He X., Bai J. (2013). Progress in the pathogenesis of pterygium. Curr. Eye Res..

[29] John-Aryankalayil M. (2006). Microarray and protein analysis of human pterygium. Mol. Vis..

[30] Livezeanu C., Crǎiţoiu M.M.C., Mǎnescu R., Mocanu C., Crǎiţoiu Ş. (2011). Angiogenesis in the pathogenesis of pterygium. Rom. J. Morphol. Embryol..

[31] Xue C., Chen Y., Huang Z., Ge Y., Wang H., Wang J. (2014). EphB4 expression in pterygium is associated with microvessel density. Int. J. Clin. Exp. Med..

[32] Xue C., Huang Z., Wang J., Dong Y., Zhou X. (2009). EphrinB2 and EphB4 expression in pterygia: New insights and preliminary results. Can. J. Ophthalmol..

[33] Mergler S., Pleyer U. (2007). The human corneal endothelium: New insights into electrophysiology and ion channels. Prog. Retin. Eye Res..

[34] Ojima T. (2006). EphrinA1 inhibits vascular endothelial growth factor-induced intracellular signaling and suppresses retinal neovascularization and blood-retinal barrier breakdown. Am. J. Pathol..

[35] White A.I., Sabater A.L. (2019). Current strategies for human corneal endothelial regeneration. Regen. Med..

[36] Giorgio C., Mohamed I.H., Flammini L., Barocelli E., Incerti M., Lodola A., Tognolini M. (2011). Lithocholic Acid Is an Eph-ephrin Ligand Interfering with Eph-kinase Activation. PLoS ONE.

[37] Azar D.T. (2006). Corneal angiogenic privilege: Angiogenic and antiangiogenic factors in corneal avascularity, vasculogenesis, and wound healing (An American Ophthalmological Society Thesis). Trans. Am. Ophthalmol. Soc..

[38] Sharif Z., Sharif W. (2019). Corneal neovascularization: Updates on pathophysiology, investigations & management. Rom. J. Ophthalmol..

[39] Abdelfattah N.S. (2015). Clinical correlates of common corneal neovascular diseases: A literature review. Int. J. Ophthalmol..

[40] Lee P., Wang C.C., Adamis A.P. (1998). Ocular neovascularization: An epidemiologic review. Surv. Ophthalmol..

[41] Alipour F., Khaheshi S., Soleimanzadeh M., Heidarzadeh S. (2017). Contact lens-related complications: A review. J. Ophthalmic Vis. Res..

[42] Papas E. (2006). Corneal vascularisation and contact lenses. Arch. Soc. Esp. Oftalmol..

[43] Gupta D., Illingworth C. (2011). Treatments for corneal neovascularization: A review. Cornea.

[44] Ellenberg D. (2010). Novel aspects of corneal angiogenic and lymphangiogenic privilege. Prog. Retin. Eye Res..

[45] Brantley-Sieders D., Chen J. (2004). Eph receptor tyrosine kinases in angiogenesis: From development to disease. Angiogenesis.

[46] Cheng N. (2002). Blockade of EphA receptor tyrosine kinase activation inhibits vascular endothelial cell growth factor-induced angiogenesis. Mol. Cancer Res..

[47] Kuijper S., Turner C.J., Adams R.H. (2007). Regulation of Angiogenesis by Eph–Ephrin Interactions. Trends Cardiovasc. Med..

[48] Kenyon B.M., Voest E.E., Chen C.C., Flynn E., Folkman J., D’Amato R.J. (1996). A model of Angiogenesis in the Mouse Cornea. Investig. Ophthalmol. Vis. Sci..

[49] Maekawa H. (2003). Ephrin-B2 Induces Migration of Endothelial Cells through the Phosphatidylinositol-3 Kinase Pathway and Promotes Angiogenesis in Adult Vasculature. Arterioscler. Thromb. Vasc. Biol..

[50] Hayashi S.I., Asahara T., Masuda H., Isner J.M., Losordo D.W. (2005). Functional ephrin-B2 expression for promotive interaction between arterial and venous vessels in postnatal neovascularization. Circulation.

[51] Uyen Huynh-Do TO D., Vindis C., Liu H., Cerretti D., McGrew J., Enriquez M., Chen J. (2002). Ephrin-B1 transduces signals to activate integrin-mediated migration, attachment and angiogenesis. J. Cell Sci..

[52] Pandey A., Shao H., Marks R.M., Polverini P.J., Dixit V.M. (1995). Role of B61, the ligand for the Eck receptor tyrosine kinase, in TNF-α-induced angiogenesis. Science.

[53] Lindauer M., Hochhaus A. (2018). Dasatinib. Recent Results Cancer Res..

[54] He S. (2005). Soluble EphB4 regulates choroidal endothelial cell function and inhibits laser-induced choroidal neovascularization. Investig. Ophthalmol. Vis. Sci..

[55] Davies M.H., Zamora D.O., Smith J.R., Powers M.R. (2009). Soluble ephrin-B2 mediates apoptosis in retinal neovascularization and in endothelial cells. Microvasc. Res..

[56] Zamora D.O., Davies M.H., Planck S.R., Rosenbaum J.T., Powers M.R. (2005). Soluble forms of EphrinB2 and EphB4 reduce retinal neovascularization in a model of proliferative retinopathy. Investig. Ophthalmol. Vis. Sci..

[57] Ehlken C. (2011). Therapeutic interference with EphrinB2 signalling inhibits oxygen-induced angioproliferative retinopathy. Acta Ophthalmol..

[58] Brar M., Cheng L., Yuson R., Mojana F., Freeman W.R., Gill P.S. (2010). Ocular safety profile and intraocular pharmacokinetics of an antagonist of EphB4/EphrinB2 signalling. Br. J. Ophthalmol..

[59] Tsang S.H., Sharma T. (2018). Drug-induced retinal toxicity. Adv. Exp. Med. Biol..

[60] Horita S. (2019). Species differences in ocular pharmacokinetics and pharmacological activities of regorafenib and pazopanib eye-drops among rats, rabbits and monkeys. Pharmacol. Res. Perspect..

[61] Kim Y.C. (2020). Gelling hypotonic polymer solution for extended topical drug delivery to the eye. Nat. Biomed. Eng..

[62] Zimmermann T. (2018). Topical administration of regorafenib eye drops: Phase I dose-escalation study in healthy volunteers. Br. J. Clin. Pharmacol..

[63] Chu M., Zhang C. (2018). Inhibition of angiogenesis by leflunomide via targeting the soluble ephrin-A1/EphA2 system in bladder cancer. Sci. Rep..

[64] Dobrzanski P. (2004). Antiangiogenic and Antitumor Efficacy of EphA2 Receptor Antagonist. Cancer Res..

[65] Janes P.W., Vail M.E., Gan H.K., Scott A.M. (2020). Antibody targeting of eph receptors in cancer. Pharmaceuticals.

[66] Tang F.H.F., Davis D., Arap W., Pasqualini R., Staquicini F.I. (2020). Eph receptors as cancer targets for antibody-based therapy. Adv. Cancer Res..

